# Case of Cancer of the Pylorus

**Published:** 1858-05

**Authors:** Ralph N. Isham

**Affiliations:** Chicago


ARTICLE II.

### CASE OF CANCER OF THE PYLORUS.

BY RALPH N. ISHAM, M. D., CHICAGO.

J. O., a Prussian by birth, thirty-two years of age, single, of temperate habits, has, with the exception of two attacks of rheumatism, always enjoyed good health. No hereditary tendency to disease traceable; father and mother still living. His occupation has been that of house painter, and recently a fruit dealer.

*Previous History.*—The first indication he had of his present disorder was in November last, when he vomited after eating pork or anything sour, and to such an extent did this symptom annoy him, in the space of a few weeks, that the stomach would only retain beef, soup, coffee and tea. On the 1st of January, after drinking two glasses of beer, and feeling a disagreeable sense of nausea, he procured from a druggist an emetic powder, which produced violent emesis. From that day he was unable to work, and since the 10th has been confined to his bed. He has had discharges of a black fœtid character several times recently, and has vomited "coffee-grounds" material once. This symptom of melæna has only appeared when preceded by violent efforts at vomiting, or the use of cathartic medicine. The ordinary stools have consisted of hard scybala. He states that he has felt a tenderness in the epigastric region for three months; that his attention was first called to it after a "severe vomiting spell;" that it has since increased until the weight of the bed-clothes causes pain.

*Present Condition, March 11th,* 1858.—He has to assume the semi-erect position on account of local tenderness, and complains of occasional sharp pains through the abdomen; is very weak; the act of vomiting is not accompanied with any voluntary effort; has a sallow complexion of skin, with a somewhat anxious expression of countenance; complains of flatulence; the food is rejected by the stomach within half an hour after eating, unless of a fluid character, and in very small quantities; the urine is scanty and loaded with urates, presenting that pink variety of uric acid deposit, regarded by many as characteristic of constitutional malignant disease; bowels are constipated; pulse 80; tongue coated with a dirty white fur; complete loss of appetite, with imsomnia. There is in the epigastric region, midway between the cartilages of the false ribs, a hard tumor the size of a billiard ball, which transmits the pulsations of the aorta, but gives no thrill or lateral dilatation; dulness on percussion over the stomach; no enlargement of the liver or spleen; nutrition moderate.

*Diagnosis.*—Scirrhus of the pylorus.

Tuesday, March 20th. Patient has been a little better yesterday; has considerable fever, and a miliary eruption covers the body; complains of a "gnawing and burning" sensation of the stomach and throat, with insatiable thirst; vomiting still continues, but not to same extent as before; the patient considerably emaciated; the countenance is more anxious; œdema of the feet first noticed to-day; the tumor and tenderness of the epigastrium are still present.

My friend, Dr. Denniston, and also Dr. Wagner, who kindly prescribed for the patient during his late illness, agree with me in the diagnosis made.

The patient died, April 10th, but for many days previously had been comparatively free from pain.

*Autopsy*, 12*th.*—Twenty-six hours post-mortem. On section of the abdomen the stomach presented a dull, anæmic appearance, and was thickened in its structure. The pylorus was involved in a crude cancerous mass, of a friable, lardaceous, and half-suppurating character, the size of an orange; all the coats were lost in the tumor. The peritoneum and liver were adherent where normally in contact. The intestines were partially filled with scybala. No effusion in the abdomen.

The examination was not extended to other organs, through respect to the wishes of friends.

That the mucous coat was the last which suffered erosion from this disease is proved by the fact that the coffee-grounds vomit did not appear until a late date, as also the gnawing pain and excessive thirst, accompaniments of the inflamed mucous membrane; whilst the acute pains, symptomatic of disease of the outer coat, were also amongst the last distressing symptoms; we are forced to conclude, that the muscular coat was the first attacked, and not the mucous, according to the pathological rule urged by many recent writers. It is whilst the serous coat is undergoing disorganization that the patient suffers the acutest pain; and when the local tenderness is excessive, it may account for the adhesive inflammation, as in this case, though very common in both cancer and *ulcer* of the stomach. That many, if not all, of the prominent symptoms in this case might have been those of gastric ulcer, I would urge as a rule, that *it is never safe to pronounce a diagnosis of gastric cancer unless the tumor can be distinctly felt.*

One of the diagnostic marks between these two diseases is the position. Whilst the favorite seat of the malignant disease is the pylorus, in the proportion of 219 cases out of 360, that of the ulcer is only 25 out of 360, in a situation which might be mistaken for the pylorus; the greater number, 177, being found on the posterior surface.

In regard to age, 600 cases afforded an average of 50 years, and at 32 (that of this subject), as compared to ulcer of the stomach, the relation is as $31\frac{2}{3}$ to 49 in 100 cases. The liability to this disease of the male over that of the female is double, up to the age of 60 years.

The cachectic appearance, which is justly considered a strong symptom in malignant disease when accompanied with emaciation, is not apt to be confounded with simple jaundice, yet, contrary to the general supposition, may be imitated by ulcer of the stomach to that degree as to defy distinction, and, of course, is only valuable in connection with *other* signs.

---

ARTICLE III.

